# Enhancing Anti-SARS-CoV-2 Neutralizing Immunity by Genetic Delivery of Enveloped Virus-like Particles Displaying SARS-CoV-2 Spikes

**DOI:** 10.3390/vaccines11091438

**Published:** 2023-08-31

**Authors:** Yongping Yang, Wing-Pui Kong, Cuiping Liu, Tracy J. Ruckwardt, Yaroslav Tsybovsky, Lingshu Wang, Shuishu Wang, Daniel W. Biner, Man Chen, Tracy Liu, Jonah Merriam, Adam S. Olia, Li Ou, Qi Qiu, Wei Shi, Tyler Stephens, Eun Sung Yang, Baoshan Zhang, Yi Zhang, Qiong Zhou, Reda Rawi, Richard A. Koup, John R. Mascola, Peter D. Kwong

**Affiliations:** 1 Vaccine Research Center, National Institute of Allergy and Infectious Diseases, National Institutes of Health, Bethesda, MD 20892, USA; yongpiny@niaid.nih.gov (Y.Y.);; 2 Vaccine Research Center Electron Microscopy Unit, Cancer Research Technology Program, Frederick National Laboratory for Cancer Research, Frederick, MD 20701, USA

**Keywords:** DNA vaccine, enveloped virus-like particle, eVLP, genetic delivery, immunization, Newcastle disease virus-like particle, neutralizing response, SARS-CoV-2 spike, T cell

## Abstract

New vaccine delivery technologies, such as mRNA, have played a critical role in the rapid and efficient control of SARS-CoV-2, helping to end the COVID-19 pandemic. Enveloped virus-like particles (eVLPs) are often more immunogenic than protein subunit immunogens and could be an effective vaccine platform. Here, we investigated whether the genetic delivery of eVLPs could achieve strong immune responses in mice as previously reported with the immunization of in vitro purified eVLPs. We utilized Newcastle disease virus-like particles (NDVLPs) to display SARS-CoV-2 prefusion-stabilized spikes from the WA-1 or Beta variant (S-2P or S-2Pᵦ, respectively) and evaluated neutralizing murine immune responses achieved by a single-gene-transcript DNA construct for the WA-1 or Beta variant (which we named S-2P-NDVLP-1T and S-2Pᵦ-NDVLP-1T, respectively), by multiple-gene-transcript DNA constructs for the Beta variant (S-2Pᵦ-NDVLP-3T), and by a protein subunit–DNA construct for the WA-1 or Beta variant (S-2P-TM or S-2Pᵦ-TM, respectively). The genetic delivery of S-2P-NDVLP-1T or S-2Pᵦ-NDVLP-1T yielded modest neutralizing responses after a single immunization and high neutralizing responses after a second immunization, comparable to previously reported results in mice immunized with in vitro purified S-2P-NDVLPs. Notably, genetic delivery of S-2Pᵦ-NDVLP-3T yielded significantly higher neutralizing responses in mice after a second immunization than S-2Pᵦ-NDVLP-1T or S-2Pᵦ-TM. Genetic delivery also elicited high spike-specific T-cell responses. Collectively, these results indicate that genetic delivery can provide an effective means to immunize eVLPs and that a multiple-gene transcript eVLP platform may be especially efficacious and inform the design of improved vaccines.

## 1. Introduction

The SARS-CoV-2 pandemic highlights an urgent need for vaccine platforms that can efficiently control viral outbreaks [1,2]. It is important that vaccines can be rapidly and efficiently made available to large populations and that protective immune responses are achieved after vaccination. Enveloped virus-like particles (eVLPs) show promise as a potential vaccine platform to combat infectious diseases because they structurally and antigenically mimic the authentic virus but lack the viral genome [3,4,5,6,7,8]. eVLPs are different from inactivated virus vaccines, which contain the viral genome and are usually made non-infectious by exposure to chemical or physical inactivating agents. Such chemical or physical treatment, such as the most commonly used inactivating agent, formalin, can induce irreversible changes in viral antigens, resulting in poor immunogenicity and weak cell-mediated and mucosal immune responses [9]. Multiple studies have shown that repetitive display of antigenic epitopes on the surface of eVLPs that range in size from ~20 to ~200 nm can facilitate optimal migration to lymph nodes and uptake by antigen-presenting cells, particularly dendritic cells (DCs). Enhanced uptake and processing by DCs enable eVLPs to induce powerful immune responses [3,4,10,11,12]. Despite this, the production of eVLPs has remained challenging. For example, the current eVLP purification process is intricate and unscalable [3,4,10,13,14,15]. The genetic delivery of eVLP through immunization with a DNA construct (DNA vaccine platform) or with a synthetic mRNA (mRNA vaccine platform) can bypass the eVLP purification process. DNA and mRNA vaccine platforms have proven to be rapidly scalable and available for the vaccination of large populations [16,17].

To circumvent the challenges of in vitro eVLP preparation, we immunized mice with eVLPs displaying SARS-CoV-2 spikes through genetic delivery with a DNA vaccine platform [18] and evaluated if genetic delivery of eVLPs could elicit strong humoral immune responses in mice as previously reported from immunization with in vitro purified eVLPs [6]. We compared humoral immune responses induced by a single-gene-transcript eVLP vaccine platform, by a multiple-gene-transcript eVLP vaccine platform, and by a protein subunit vaccine platform, the latter of which had the same sequence as that utilized in a SARS-CoV-2 mRNA vaccine [17,19], expressed as a membrane-bound form, which has shown success in combating COVID-19. Our study demonstrates that the genetic delivery of eVLPs could provide a rapid and efficient means to deliver eVLPs and that a different vaccine platform may have a different mechanism of inducing neutralizing antibodies, which could potentially inspire new vaccines that elicit strong and durable neutralizing immunity.

## 2. Materials and Methods

### 2.1. Animal

BALB/c mice were obtained from Jackson Laboratories (Bar Harbor, ME, USA), and all animals were housed and cared for in accordance with American Association for Accreditation of Laboratory Animal Care standards in accredited facilities.

### 2.2. Cell Line

HEK293T cells (#CRL3216, ATCC, VA) were cultured in Dulbecco’s Modified Eagle Medium (DMEM)-formulated optimal cell growth medium containing 12% inactivated fetal bovine serum (FBS) and 100 U/mL streptomycin–penicillin (ABI Scientific, Sterling, VA, USA) at 37 °C and 5% CO_2_.

### 2.3. Preparation of DNA Constructs

DNA construct of S-2P-TM, a full-length of WA-1 SARS-CoV-2 prefusion-stabilized spike gene with two proline substitutions at residues 986–987 including spike ectodomain (S-2P, amino acid residues 1–1208) and transmembrane cytoplasm tail domain (TM, amino acid residues 1209–1273), encoded a cell-membrane-anchored S-2P (GenBank: MN908947). The DNA construct of S-2Pᵦ-TM was the Beta variant of spike (B.1.351) [20]. The DNA construct of S-2P-NDV-Ftm or S-2Pᵦ-NDV-Ftm, a chimeric gene including a SARS-CoV-2 prefusion-stabilized spike ectodomain gene (S-2P or S-2Pᵦ, amino acid residues 1–1208) fused with an NDV fusion protein (NDV-F, avian avulavirus 1, GenBank: AMQ09764) transmembrane-cytoplasmic domain gene (Ftm, amino acid residues 499–553), encoded a cell-membrane-anchored S-2P or S-2Pᵦ [6]. To make a DNA construct of a single-gene-transcript S-2P-NDVLP-1T or S-2Pᵦ-NDVLP-1T, three genes of the NDV matrix (NDV-M, amino acid residues 1–364, and avian avulavirus 1; GenBank: AMQ09763), nucleoprotein (NDV-NP, amino acid residues 2–489, and avian avulavirus 1; GenBank: AMQ09761), and S-2P-NDV-Ftm or S-2Pᵦ-NDV-Ftm were linked by two self-cleavage peptide sequences (P2A and T2A) as a monocistronic DNA molecule, which encoded a single polypeptide that was cleaved into three individual proteins of NDV-M, NDV-NP, and cell-membrane-anchored S-2P or S-2Pᵦ during post-translation processing in the cell, with exclusive self-assembly into secreted S-2P-NDVLP or S-2Pᵦ-NDVLP. Multiple-gene-transcript DNA constructs of S-2P-NDVLP-3T or S-2Pᵦ-NDVLP-3T were composed of three individual genes of NDV-M, NDV-NP, and S-2P-NDV-Ftm or S-2Pᵦ-NDV-Ftm, which directly encoded three individual proteins of NDV-M, NDV-NP, and cell-membrane-anchored S-2P or S-2Pᵦ, respectively.

To make a DNA construct of single-gene-transcript S-2P-CoV2VLP-1T, the envelope gene (E), matrix gene (M), and full-length prefusion-stabilized spike gene (S-2P-TM) from a WA-1 SARS-CoV2 isolate were linked by two self-cleavage peptide sequences (P2A and T2A) as a monocistronic DNA molecule, which encoded a single polypeptide that was cleaved into three individual proteins of E, M, and cell-membrane-anchored S-2P during post-translation processing in the cell, with exclusive self-assembly into secreted S-2P-CoV2VLP. Multiple-gene-transcript DNA constructs of S-2P-CoV2VLP-3T were composed of WA-1 SARS-CoV2 and three individual genes of E, M, and S-2P-TM, directly encoding three individual proteins of E, M, and cell-membrane-anchored S-2P, respectively. Expression control for the DNA construct of S-2P or S-2Pᵦ was achieved with the gene of the prefusion-stabilized spike ectodomain (amino acid residues 1–1208) from the SARS-CoV2 WA-1 or Beta isolate. Each gene was codon-optimized, synthesized, and cloned into a VRC8400 vector (CMV/R expression vector developed and used in house) as a plasmid DNA construct (GeneImmune Biotechnology, New York, NY, USA).

### 2.4. Cell Culture Microplate-Formatted Expression

First, 2.5 × 10^4^ log-phase HEK 293T cells in 100 μL of RealFect Expression medium (ABI Scientific, Sterling, VA, USA) per well were inoculated in a 96-well cell culture microplate (Corning Scientific, Corning, NY, USA) and allowed to grow for 24 h at 37 °C under 5% CO_2_. Immediately before transfection, 40 μL of spent medium per well was removed, and 250 ng of plasmid DNA encoding either eVLP or soluble protein in 10 μL of Opti-MEM Reduced Serum medium (Thermo Fisher Scientific, Rockville, MD, USA) was mixed with 0.75 μL of TrueFect Max transfection reagent (United Biosystems, Bethesda, MD, USA) in 10 μL of Opti-MEM Reduced Serum medium at room temperature (RT) for 15 min, and then mixed with growing cells in each well in the 96-well cell culture microplate. Transfected cells were incubated at 37 °C under 5% CO_2_ for 6–12 h, then fed with 25 μL of CelBooster medium (Cell Growth Enhancer for adherent cells, ABI Scientific) per well, with an additional 10% FBS and 3 × 100 U/mL streptomycin–penicillin. Starting from day 3 post transfection, the supernatants in the wells of a 96-well cell culture microplate were sampled, and the yields of eVLPs were measured by an eVLP ELISA assessment with a SARS-CoV-2 neutralizing antibody (S309).

### 2.5. Expression and Purification of eVLP

First 7 × 10^6^ log-phase growing HEK293T cells were seeded in a T75-cm^2^ flask (Corning Scientific) and cultured at 37 °C under 5% CO_2_ for 24 h, achieving 85% cell confluency. Prior to transfection, the spent culture medium in the flask was replaced with 8 mL of fresh RealFect expression medium, and 25 μg of plasmid DNA in 1.1 mL of serum-free Opti-MEM medium was mixed with 75 μL of TrueFect transfection reagent in 1.1 mL of serum-free Opti-MEM for 15 min at RT, then mixed with growing cells in a T75-cm^2^ flask. Transfected cells were incubated at 37 °C under 5% CO_2_ for 6–12 h, then fed with 5 mL of CelBooster medium, with an additional 10% FBS and 3 × 100 U/mL streptomycin–penicillin. Three days after transfection, the culture supernatant in transfected cell flasks was harvested, and eVLPs were purified by polyethylene glycol (PEG)-mediated precipitation and sucrose gradient ultracentrifugation method as follows. Harvested supernatant was clarified by centrifugation at 1500× *g* at 4 °C for 30 min, followed by filtration through a 0.8 μm filter, slow mixing with ¼ volume of Universal PEG Virus Precipitation Solution (ABI Scientific), and gentle shaking at 4 °C for 2 h. Then, the supernatant was incubated at 4 °C overnight. After centrifugation of the supernatant–PEG mixture at 2000× *g* for 30 min at 4 °C, the top phase solution was removed, and precipitated eVLPs in pellet form were resuspended in MES buffer (150 mL NaCl, 20 mM MES, pH 6.0) and transferred onto the top of a discontinuous sucrose gradient in a 12 mL ultracentrifuge tube (Beckman Coulter, Brea, CA, USA) with 65% sucrose on the bottom and 10% sucrose on the top and a total of 6 layers (65%, 50%, 40%, 30%, 20%, and 10%; 1.5 mL/layer), followed by ultracentrifugation at 36,000 rpm at 4 °C for 12 h (Beckman SW28.1 rotor) [6,21]. eVLPs were sedimented in 10–20% and 40% sucrose layers, as determined by eVLP ELISA assessment with a SARS-CoV-2 neutralizing antibody (S309), and stored at −80 °C for analyses. The concentration of eVLPs was measured with a Micro BCA Protein Assay kit (Themo Fisher Scientific).

### 2.6. Negative Staining Electron Microscopy

The ultracentrifugation-purified eVLP samples were applied to carbon-coated copper grids that were subjected to glow discharging immediately before the experiment. The drop volume was 4.8 µL. After a brief incubation, the drop was removed with filter paper. Three washes with buffer containing 10 mM HEPES (pH 7) and 150 mM NaCl were performed in the same manner. After the final wash, adsorbed material was negatively stained by consecutively applying three drops of 0.7% uranyl formate. Images were recorded at a nominal magnification of 57,000× using a Thermo Scientific Talos F200C electron microscope equipped with a Ceta camera and operated at 200 kV. The pixel size was 0.25 nm.

### 2.7. Preparation of Prefusion-Stabilized SARS-CoV-2 Spike-Soluble Trimeric Glycoprotein (S-2P or S-2Pᵦ)

The expression plasmid design and protein production of S-2P or S-2Pᵦ trimer protein were performed as described previously [22]. Briefly, each protein sequence was fused with an 8-mer His-tagger sequence at the carboxyl terminus, and the DNA-encoding code was optimized and synthesized by GeneImmune Biotechnology. Each His-tagged protein was expressed in 293 Freestyle cells by transient transfection for 6 days at 37 °C. On days 1 and 3 post transfection, an enriched feed medium, Cell Growth Enhancer for suspension cells (ABI Scientific, Sterling, VA, USA), was added into the culture at 5% culture volume. Following expression, the cell supernatant was collected and filtered and applied to nickel affinity resin. The protein-bound resin was washed extensively with an increasing imidazole concentration and eluted with 300 mM imidazole buffer. His-tagged HRV3c protease was added to the nickel elution and incubated overnight at 4 °C. The cleaved protein was then concentrated using spin filters and applied to a Superdex S-200 gel filtration column and equilibrated in PBS. The S-2P or S-2Pᵦ protein was eluted primarily in a single peak corresponding to a trimeric size. The peak fraction was collected, concentrated to 1 mg/mL, and flash-frozen in liquid nitrogen prior to storage at −80 °C.

### 2.8. Preparation of SARS-CoV-2 Neutralizing Antibodies

Antibody heavy-chain and light-chain sequences were codon-optimized, synthesized, and cloned into a VRC8400-based IgG1 vector and co-expressed by transient transfection within Expi293 cells (Thermo Fisher Scientific) according to the manufacturer’s recommendation. Briefly, 50 μg plasmid-encoding heavy-chain genes and 50 μg plasmid-encoding light-chain genes were mixed with 300 μL of Turbo293 transfection reagent (SPEED BioSystems, Gaithersburg, MD, USA) for 15 min, added to 100 mL of cells at a concentration of 2.5 × 10^6^/mL, and incubated in a shaker incubator at 120 rpm and 37 °C under 9% CO_2_. On days 1 and 3 post transfection, an enriched feed medium, AbBooster Antibody Expression Enhancer for suspension cells (ABI Scientific), was added into the culture at a 10% culture volume. After 5 days post transfection, cell culture supernatant was harvested and purified with a Protein A (GE Healthcare, Chicago, IL, USA) column. The antibody was eluted using IgG Elution Buffer (Thermo Fisher Scientific) and brought to neutral pH (7.0) with 1 M Tris-HCl (pH 8.0). Eluted antibodies were dialyzed against PBS overnight before use.

### 2.9. eVLP ELISA Assessment

First, 96-well ELISA plates (Nunc Maxisorp, Thermo Fisher Scientific) were coated with 100 μL/well of lectin (Galanthus Nivalis, SIGMA, St. Louis, MO, USA) at a concentration of 5 μg/mL in PBS overnight at 4 °C, followed by blocking with a standard blocking buffer, 1% BSA, and 0.05% Tween in PBS. Following washing, 33 μL of eVLP expression supernatant was mixed with 67 μL of PBS per well and incubated in precoated lectin plates for 1.5 h at RT. Following washing, 100 μL/well of SARS-CoV-2 neutralizing antibodies (S309 or others) at a concentration of 10 μg/mL was incubated in the plates for 50 min at RT. Then, bound antibodies were detected by incubation with a goat anti-human IgG Fc fragment–horseradish peroxidase (HRP) conjugate (Jackson Immunoresearch, West Grove, PA, USA) at 1 × 10^4^ dilution in blocking buffer, as per the manufacture’s instructions, for 30 min at RT. Following washing, the bound anti-human IgG Fc fragment–HRP conjugate was detected with HRP substrate 3,5,3′5′-tetramethylbenzidine (TMB) (BioFX TMB, SurModics, Eden Prairie, MN, USA) for 10 min at RT. The reaction was stopped with 0.5 N sulfuric acid. The optical density at 450 nm (OD450) was read with a spectrophotometer (SpectroPlus, Molecular Devices, San Jose, CA, USA).

### 2.10. eVLP Antigenic Analysis

First, 96-well ELISA plates (Nunc Maxisorp, Thermo Fisher Scientific) were coated with 100 μL per well of lectin (Galanthus Nivalis, SIGMA) at a concentration of 5 μg/mL in PBS overnight at 4 °C, followed by blocking with a standard blocking buffer, 1% BSA, and 0.05% Tween in PBS. Following washing, 100 μL per well of purified eVLP at a concentration of 25 μg/mL or S-2P or S-2Pᵦ trimer protein at a concentration of 1 μg/mL was incubated in precoated lectin plates for 1.5 h at RT. Following washing, 100 μL per well of serially diluted SARS-CoV-2 neutralizing antibodies at a concentrations ranging from 0.00064 μg/mL to 50 μg/mL was incubated in the plates for 50 min at RT. Then, bound antibodies were detected by incubation with a goat anti-human IgG Fc fragment–HRP conjugate (Jackson Immunoresearch, PA) at 1 × 10^4^ dilution in blocking buffer for 30 min at RT. Following washes, the bound anti-human IgG Fc fragment–HRP conjugate was detected with the HRP substrate of BioFX TMB for 10 min at RT. The reaction was stopped with 0.5 N sulfuric acid. The optical density (OD450) was read at 450 nm with a spectrophotometer (SpectroPlus, Molecular Devices). OD450 values versus serial dilution of SARS-CoV-2 neutralizing antibodies was illustrated using GraphPad Prism v8 software (GraphPad Software, San Diego, CA, USA).

### 2.11. Mouse Immunization

Female BALB/c mice, aged 8–12 weeks (Jackson Laboratories, Bar Harbor, ME, USA), were immunized intramuscularly by injection and electroporation (BTX AgilePulse, Holliston, MA, USA) with indicated doses of plasmid DNA at week(s) 0, 4 (*n* = 10 per group), or 20 (*n* = 5 per group), respectively. The mice were injected with 100 μL of plasmid DNA into the medial large muscle (m. vastus medialis) of each hind limb, followed by electroporation. Two doses comprising different amounts of plasmid DNA were used: a high dose of 50 μg per immunization per mouse and a low dose of 10 μg per immunization per mouse. For immunization of S-2Pᵦ-NDVLP-3T, three individual plasmid DNAs were mixed in the same plasmid molar ratio. Mice were bled at week(s) 0, 2, 6, 10, 14, 18, and 22, and sera were collected accordingly for serological analysis.

### 2.12. SARS-CoV-2 Spike-Specific Serum IgG Assay

WA-1 or Beta SARS-CoV-2 spike-specific IgG titers were assessed with an antibody titration ELISA as described previously [6]. Briefly, ELISA plates (Thermo Fisher Scientific, 442404) were coated with 1 µg/mL of SARS-CoV-2 WA-1 S-2P or S-2Pᵦ in PBS (pH 7.4) at 4 °C for 16 h. The plates were washed with PBST three times, then blocked with 5% skim milk in 1 × PBST at RT for 2 h. The sera were diluted by 100-fold in 5% skim milk in PBST. A further serial 4-fold dilution for week-0 and week-2 sera or 6-fold dilution for week-6 sera was applied to the 100-fold dilution preparations. The plates were incubated with diluted sera at RT for 1 h. The HRP-conjugated anti-mouse secondary antibody (Thermo Fisher Scientific, 1/2000 dilution) was used to detect the antibody responses. The endpoint titers were calculated as the dilution that yielded an optical density equivalent to 4× background (secondary antibody alone).

### 2.13. Pseudovirus Neutralization Titer Assay

WA-1 or Beta SARS-CoV-2 neutralization titers were measured using a lentivirus-based SARS-CoV-2 pseudovirus assay. Pseudoviruses were generated by co-transfection of transducing plasmid pHR’ CMV-Luc encoding a luciferase reporter, lentivirus packaging plasmid pCMVd8.2, and a TMPRSS2 plasmid and S plasmids from SARS-CoV-2 WA-1 isolate (GenBank: MN908947.3), and B.1.351 isolate (Beta) into HEK293T/17 cells (ATCC CRL-11268) using Lipofectamine 3000 transfection reagent (L3000-001, ThermoFisher Scientific) [17,20,23]. Heat-inactivated serum was mixed with the titrated pseudoviruses, incubated, then added to preplated 293T-ACE2 cells (provided by Dr. Michael Farzan) in triplicate. Following a 2 h incubation, wells were replenished with 150 µL of fresh medium. Then, 72 h later, the cells were lysed, and the luciferase activity was recorded in relative light units (RLUs). Percent neutralization and neutralization ID_50_s were calculated using GraphPad Prism 9.0.2.

### 2.14. Intracellular Cytokine Staining and Flow Cytometry Assay

Mouse splenocytes were obtained by using a gentleMACS tissue dissociator (Miltenyi Biotec, Gaithersburg, MD, USA), followed by 70 µm filtration and density gradient centrifugation using Fico/Lite-LM medium (Atlanta Biologicals, Flowery Branch, GA, USA). Cells from each mouse spleen were resuspended in R10 media (RPMI 1640 supplemented with pen–strep antibiotic, 10% HI-FBS, Glutamax, and HEPES) and incubated for 6 h at 37 °C with protein transport inhibitor cocktail (eBioscience, San Diego, CA, USA) under three conditions: no peptide (DMSO only) stimulation or stimulated with one of two spike peptide pools (S1 and S2 peptide pools, 85% pure, JPT Peptide Technologies, Berlin, Germany). Peptide pools were used at a final concentration of 2 µg/mL for each peptide. After stimulation, cells were washed with PBS prior to staining with LIVE/DEAD Fixable Blue Dead Cell Stain (Invitrogen) for 20 min at RT, then washed in FC buffer (PBS supplemented with 2% HI-FBS and 0.05% NaN_3_). Cells were then resuspended in BD Fc Block (clone 2.4G2) for 5 min at RT prior to staining with a surface stain cocktail containing the following antibodies purchased from BD and Biolegend: I-A/I-E (M5/114.15.2) PE, CD8a (53-6.7) BUV805, CD44 (IM7) BUV395, CD62L (MEL-14) BV605, and CD4 (RM4-5) BV480 in brilliant stain buffer (BD). After a 15 min incubation at RT, cells were washed with FC buffer, then fixed and permeabilized using a BD Cytofix/Cytoperm fixation/permeabilization solution kit according to manufacturer’s instructions. Cells were washed using perm/wash solution and stained with Fc Block (5 min at RT), followed by intracellular staining (30 min at 4 °C) using a cocktail of the following antibodies purchased from BD, Biolegend, or eBioscience: CD3e (17A2) BUV737, IFN-γ (XMG1.2) BV650, TNF-α (MP6-XT22) BV711, IL-2 (JES6-5H4) BV421, IL-4 (11B11) Alexa Fluor 488, IL-5 (TRFK5) APC, and IL-13 (eBio13A) PE-Cy7 in 1× perm/wash diluted with brilliant stain buffer. Finally, cells were washed in perm/wash solution and resuspended in 0.5% PFA-FC stain buffer prior to running on a Symphony A5 flow cytometer (BD Biosciences). Analysis was performed using FlowJo software, version 10.6.2, according to the gating strategy. Background cytokine expression in the no-peptide condition was subtracted from that measured in the S1 and S2 peptide pools for each individual mouse.

### 2.15. Statistical Analysis

All experimental data were subjected to statistical analyses using GraphPad Prism v9 software. IgG endpoint titers or neutralization ID_50_ values were calculated using non-linear dose–response regression analysis. Multiple comparisons between groups were calculated using a two-way ANOVA test in GraphPad Prism v9. A *t* test was used to test differences between the groups. For T-cell assay results, Kruskal–Wallis and post hoc Mann–Whitney U tests with Bonferroni correction and two-way repeated-measures ANOVA tests with multiple post hoc comparisons and Dunnett’s correction were used to detect significant differences between the groups.

## 3. Results

### 3.1. Design and Characterization of Genetic DNA Constructs Encoding eVLPs Displaying SARS-CoV-2 Spikes

**Figure 1:**
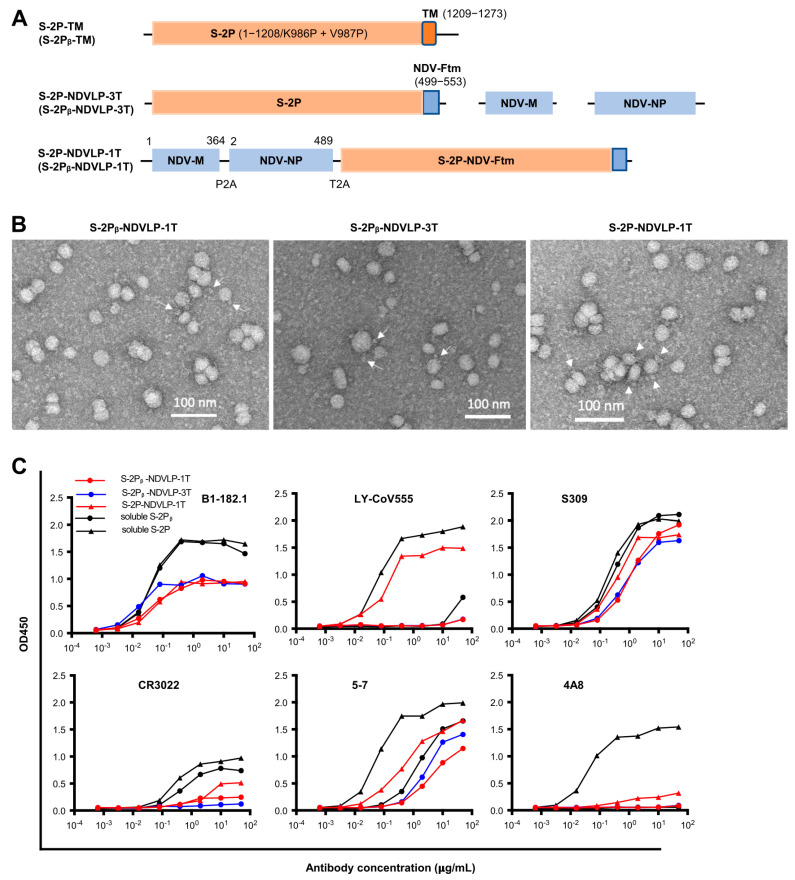
Generation and characterization of genetic DNA constructs encoding enveloped virus-like particles (eVLPs) displaying SARS-CoV-2 spikes. (**A**) The top linear diagram represents a protein subunit construct encoding S-2P-TM (WA-1 isolate) or S-2Pᵦ-TM (Beta isolate), a full-length SARS-CoV-2 spike gene composed of a prefusion-stabilized ectodomain with two proline mutations (S-2P or S-2Pᵦ (orange)) and a transmembrane cytoplasm tail domain (TM, red). The middle three linear diagrams represent the multiple-gene-transcript constructs (S-2P-NDVLP-3T or S-2Pᵦ-NDVLP-3T) composed of an NDV matrix gene (NDV-M, blue), nucleoprotein gene (NDV-NP, blue), and chimeric gene S-2P-NDV-Ftm or S-2Pᵦ-NDV-Ftm, a chimeric construct encoding SARS-CoV-2 a prefusion-stabilized spike ectodomain (S-2P, orange) fused with an NDV fusion protein transmembrane cytoplasm tail domain (NDV-Ftm, blue). The lower linear diagram represents a single-gene-transcript construct (S-2P-NDVLP-1T or S-2Pᵦ-NDVLP-1T) in which three genes, NDV-M, NDV-NP, and chimeric S-2P-NDV-Ftm or S-2Pᵦ-NDV-Ftm, were linked together by two self-cleaving peptide sequences (P2A and T2A). (**B**) Structural and morphological characterization of S-2P-NDVLP or S-2Pᵦ-NDVLP by negative staining transmission electron microscopy, showing well-defined eVLP (20–40 nm) with scattered and protruded spikes (~10 nm) on the surfaces of eVLPs. (**C**) Antigenicity characterization of S-2P-NDVLP or S-2Pᵦ-NDVLP with a panel of SARS-CoV-2 spike-specific neutralizing antibodies of B1-182.1, LY-CoV555, S309, CR3022, 4A8, and 5-7. The amount of soluble S-2P or S-2Pᵦ used at a concentration of 1 µg/mL and that amount of S-2P-NDVLP or S-2Pᵦ-NDVLP used at a concentration of 25 µg/mL are presented. Each assay was duplicated (*n* = 2).

Two types of eVLPs, Newcastle disease virus-like particle (NDVLP) and SARS-CoV-2 virus-like particle (CoV2VLP), displaying SARS-CoV-2 prefusion-stabilized spike, named S-2P-NDVLP [6] and S-2P-CoV2VLP [24], respectively, were chosen for the current study and compared in parallel with a cell-membrane-anchored full-length prefusion-stabilized spike (S-2P-TM) (Figure 1A and Figure S2A). The amino acid sequence of S-2P-TM was the SARS-CoV-2 spike WA-1 isolate [6], which is identical to the amino acid sequence of the Moderna mRNA-1273 vaccine. At the time that this study was started, the global SARS-CoV-2 variant of concern was B.1.351 (Beta variant); therefore, the Beta SARS-CoV-2 prefusion-stabilized spike (S-2Pᵦ) was also included in this study. For genetic delivery, plasmid DNA encoding S-2P-TM or S-2P-NDVLP was delivered into muscle cells by intramuscular injection followed by electroporation [16,18,25]. To ensure in vivo production of unique eVLPs, a single-gene-transcript platform (1T) was designed in which three genes were linked together with self-cleavage linkers (P2A and T2A) as a single gene that produced a single mRNA poly transcript [26,27], enabling host cells to produce secreted eVLPs in vivo. In contrast, the multiple-gene-transcript platform (3T) in which three individual genes produced three individual mRNA transcripts separately resulted in production of both cell-membrane-anchored spikes and secreted eVLPs in vivo (Figure S1).

To engineer a DNA construct encoding S-2P-NDVLP or S-2Pᵦ-NDVLP, a chimeric DNA construct encoding S-2P-NDV-Ftm or S-2Pᵦ-NDV-Ftm, a SARS-CoV-2 spike ectodomain fused with a transmembrane/cytoplasm tail domain (tm) of Newcastle disease virus fusion glycoprotein (NDV-F), was made (Figure 1A) [6,21]. The genetic delivery of a DNA construct encoding S-2P-NDV-Ftm or S-2Pᵦ-NDV-Ftm produced cell-membrane-anchored S-2P or S-2Pᵦ on the surface of host cell [6]. To engineer a single-gene-transcript DNA construct of S-2P-NDVLP-1T or S-2Pᵦ-NDVLP-1T, three individual genes encoding an NDV matrix (NDV-M), nucleoprotein (NDV-NP), and S-2P-NDV-Ftm or S-2Pᵦ-NDV-Ftm were linked together, along with two self-cleavage peptide sequences, P2A and T2A (Figure 1A). After genetic delivery, this single-gene-transcript DNA construct produced a single-polypeptide complex, which was post-translationally cleaved into three individual proteins of NDV-M, NDV-NP, and S-2P-NDV-Ftm or S-2Pᵦ-NDV-Ftm in the same host cell, which further self-assembled into secreted S-2P-NDVLPs or S-2Pᵦ-NDVLPs (Figure S1) [26,27]. The multiple-gene-transcript DNA constructs, S-2P-NDVLP-3T or S-2Pᵦ-NDVLP-3T, comprised three individual genes encoding NDV-M, NDV-NP, and S-2P-NDV-Ftm or S-2Pᵦ-NDV-Ftm, respectively (Figure 1A). Genetic codelivery of those three individual genes resulted in various gene distribution combinations among individual host cells. The cells receiving S-2Pᵦ-NDV-Ftm gene with or without NDV-NP gene produced cell-membrane-anchored S-2Pᵦ on the host cell surface, and those receiving both S-2Pᵦ-NDV-Ftm and NDV-M genes with or without NDV-NP gene produced secreted S-2Pᵦ-NDVLP. Thus, the genetic delivery of S-2Pᵦ-NDVLP-3T produced both cell-membrane-anchored S-2Pᵦ and secreted S-2Pᵦ-NDVLP in vivo (Figure S1). In parallel, the DNA constructs of S-2P-CoV2VLP-1T and S-2P-CoV2VLP-3T were generated with three individual SARS-CoV-2 genes encoding envelope protein (E), matrix protein (M), and S-2P-TM (Figure S2A). In addition, the DNA constructs encoding soluble S-2P and S-2Pᵦ were prepared as in vitro expression controls.

To evaluate the expression of eVLPs with DNA constructs, each construct was individually transfected into HEK 293T cells with the same weight. For the multiple-gene-transcript constructs, each DNA plasmid was mixed at same molar ratio. The yield of secreted eVLPs in the cell culture supernatants was evaluated by detection of S-2P or S-2Pᵦ with an eVLP capture ELISA using SARS-CoV-2 neutralizing antibody S309 (Figure S2B,C). Overall, the yields of soluble S-2P and S-2Pᵦ were similar. The eVLP levels expressed with S-2P-NDVLP-1T or S-2Pᵦ-NDVLP-1T DNA constructs were similar, at about 47% of the expression levels for soluble S-2P or S-2Pᵦ (Figure S2B) and about 80% of the eVLP expression levels of S-2P-NDVLP-3T or S-2Pᵦ-NDVLP-3T (Figure S2B,C). Also, the eVLP levels expressed with S-2P-NDVLP-3T or S-2Pᵦ-NDVLP-3T DNA constructs were similar. Interestingly, the eVLP level expressed with the S-2P-CoV2VLP-1T DNA construct was 1.8-fold higher than that expressed with the three S-2P-CoV2VLP-3T DNA constructs. In addition, yields of S-2P-NDVLP were significantly higher than those of S-2P-CoV2VLP (Figure S2B,C). Therefore, S-2P-TM, S-2Pᵦ-TM, S-2P-NDVLP-1T, S-2Pᵦ-NDVLP-1T, and S-2Pᵦ-NDVLP-3T DNA constructs were selected for further study.

To characterize the structure and antigenicity of S-2P-NDVLP and S-2Pᵦ-NDVLP expressed from the DNA constructs, the secreted eVLPs in cell culture supernatants were purified by sucrose gradient ultracentrifugation [6]. Negative stain electron microscopy (EM) revealed that purified S-2P-NDVLP, S-2Pᵦ-NDVLP, and S-2P-CoV2VLP were roughly spherical, with diameters ranging from 20 nm to 40 nm (as measured to the outer lipid bilayer) and scattered and protruded spikes (~10 nm) on the surface of eVLPs. EM also revealed that S-2P-NDVLP, S-2Pᵦ-NDVLP, and S-2P-CoV2VLP were structurally alike (Figure 1B and Figure S2D). Variation in observed eVLP sizes was likely due to the variation in the distribution of the protein components (membrane-anchored S-2P/S-2Pᵦ, matrix protein, and nucleoprotein/envelope protein) incorporated into the eVLPs. The antigenic profile of S-2P-NDVLP or S-2Pᵦ-NDVLP was measured with an antibody-binding analytical ELISA against a panel of SARS-CoV-2 neutralizing antibodies, including the RBD-specific B1-182.1, LY-CoV555, S309, and CR3022, as well as the NTD-specific 4A8 and 5-7. The profiles of eVLPs binding to neutralizing antibodies, except 4A8, were, in general, comparable to those of soluble S-2P or S-2Pᵦ (Figure 1C), indicating that S-2P-NDVLP or S-2Pᵦ-NDVLP was antigenically similar to S-2P or S-2Pᵦ. The weak binding of S-2P-NDVLP or S-2Pᵦ-NDVLP to NTD-specific antibody 4A8 suggested that the 4A8 epitope in the NTD on the surfaces of eVLPs was likely not fully accessible. Similar observations have been reported for HIV chimeric VLPs [28]. Overall, these results demonstrate that S-2Ps or S-2Pᵦs were present on the surface of eVLPs with structural and antigenic characteristics comparable to those of SARS-CoV-2 VLPs, soluble S-2P or S-2Pᵦ (Figure 1B,C and Figure S2D), suggesting that S-2P-NDVLP or S-2Pᵦ-NDVLP structurally and antigenically mimicked authentic SARS-CoV-2 virus.

### 3.2. Genetic Delivery of S-2P-NDVLP-1T or S-2Pᵦ-NDVLP-1T Elicited Strong Anti-SARS-CoV-2 Neutralization Responses in Mice, While S-2Pᵦ-NDVLP-3T Elicited More Powerful Neutralizating Responses

**Figure 2:**
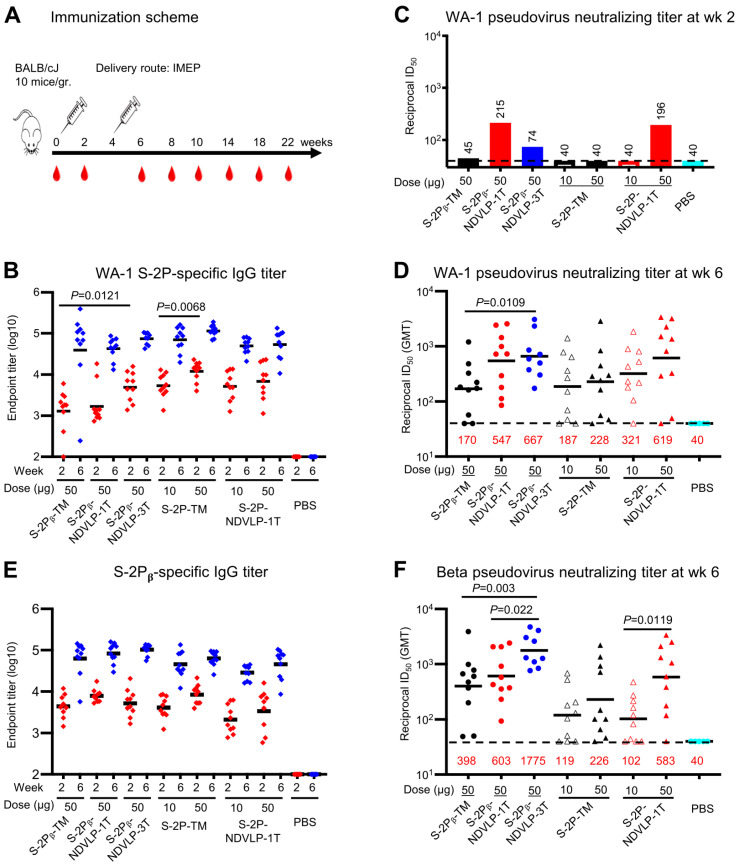
Genetic delivery of eVLPs elicited strong anti-SARS-CoV-2 pseudovirus neutralizing responses in mice. (**A**) Scheme of the genetic delivery regimen. (**B**) Genetic delivery elicited modest WA-1 spike-specific IgG titers at week 2 (red) or robust IgG titers at week 6 (blue). (**C**) After the first genetic delivery, either S-2Pᵦ-NDVLP-1T or S-2P-NDVLP-1T elicited a modest WA-1 pseudovirus neutralizing titer, while this was not the case for S-2P-TM or S-2Pᵦ-TM. S-2Pᵦ-NDVLP-3T elicited a lower WA-1 pseudovirus neutralizing titer. The sera samples from each group were group-pooled in neutralizing assays (Figure 2C). (**D**) After the second genetic delivery, WA-1 pseudovirus neutralizing titers in S-2Pᵦ-NDVLP-1T- or S-2P-NDVLP-1T-immunized groups (50-ug dose) were 3.2- or 2.7-fold higher than their counterparts, S-2Pᵦ-TM and S-2P-TM, respectively. Notably, S-2Pᵦ-NDVLP-3T induced significantly higher WA-1 pseudovirus neutralizing titers than S-2Pᵦ-TM (3.9-fold higher). (**E**) Modest Beta spike-specific IgG titers were elicited after the first genetic delivery at week 2 (red), with robust IgG titers after the second genetic delivery (week 6, blue). (**F**) After the second genetic delivery, S-2Pᵦ-NDVLP-1T and S-2P-NDVLP-1T elicited 1.5- and 2.6-fold higher Beta pseudovirus neutralization titers than S-2Pᵦ-TM and S-2P-TM, respectively. S-2Pᵦ-NDVLP-3T synergically elicited robustly higher Beta pseudovirus neutralization titers than S-2Pᵦ-TM or S-2Pᵦ-NDVLP-1T by 4.5- and 2.9-fold, respectively. A two-tailed Mann–Whitney *t* test was used to calculate the significant difference between the groups. Geometric mean titers (GMTs) in each group are marked in red font.

To evaluate if the genetic delivery of S-2P-NDVLP-1T or S-2Pᵦ-NDVLP-1T could induce high humoral immune responses as previously reported in the mice immunized with in vitro purified S-2P-NDVLP [6], BALB/c mice were immunized intramuscularly and electroporated (IMEP) with plasmid DNA constructs (Figure 2A). Five groups of mice received two injections at week 0 and week 4 with a high dose (50 μg) of the plasmid DNA construct of S-2P-NDVLP-1T, S-2Pᵦ-NDVLP-1T, S-2Pᵦ-NDVLP-3T, S-2P-TM, or S-2Pᵦ-TM. The design of the animal immunization is summarized in Table 1. Additionally, two groups received a low dose (10 μg) of a plasmid DNA construct of S-2P-TM or S-2P-NDVLP-1T. A control group received phosphate-buffered saline (PBS). Two weeks after the first genetic delivery (at week 2), WA-1 spike-specific IgG titers (ELISA endpoint titers) among all groups were modest, and the endpoint geometric mean titer (GMT) ranged from 1.5 × 10^3^ to 1 × 10^4^. The spike-specific IgG titers in mouse sera immunized with S-2Pᵦ-NDVLP-3T were significantly higher than those of S-2Pᵦ-TM-immunized mice (*p* = 0.0121), and IgG titers in mouse sera immunized with 50 μg of S-2P-TM were significantly higher than those immunized with 10 μg (*p* = 0.0068) (Figure 2B). Modest neutralizing titers against WA-1 SARS-CoV-2 pseudovirus were observed in group-pooled mouse sera (we used pooled sera to reduce the number of neutralization samples that needed to be assessed) immunized with 50 μg of S-2Pᵦ-NDVLP-1T or S-2P-NDVLP-1T (ID_50_ value of 215 or 196, respectively), and a low neutralizing titer was observed in the group-pooled mouse sera immunized with 50 μg of S-2Pᵦ-NDVLP-3T (ID_50_ value 74) (Figure 2C). No neutralization was observed in other groups, including those with 50 μg of S-2Pᵦ-TM or S-2P-TM, and all 10 μg groups. For verification of the results obtained with group-pooled mouse sera presented in Figure 2C, neutralizing titers were additionally assessed on 25 individual animal samples from week 2 and mouse groups: S-2P-TM (50 μg), S-2P-NDVLP-1T (50 μg), and PBS control (Figure S3A). Overall, the results indicate that genetic delivery of S-2P-NDVLP-1T elicits a modest pseudovirus neutralizing titer with *p* = 0.0102, which is significantly higher than that of S-2P-TM (Figure S3A).

Two weeks after the second genetic delivery (week 6), WA-1 spike-specific IgG titers increased by 10- to 35-fold relative to those from week 2, with endpoint GMT ranging from 6.5 × 10^4^ to 1 × 10^5^ (Figure 2B). No significant difference in endpoint GMT was observed among all immunized mouse groups. However, WA-1 pseudovirus neutralizing titers in the mouse sera with 50 μg of S-2P-NDVLP-1T, S-2Pᵦ-NDVLP-1T, or S-2Pᵦ-NDVLP-3T (GMT ID_50_ values ranging from 547 to 667) were 2.7- to 3.9-fold higher than those immunized with 50 μg of S-2Pᵦ-TM or S-2P-TM (GMT ID_50_ values 170 and 228, respectively) (Figure 2D). The group immunized with S-2Pᵦ-NDVLP-3T exhibited significantly higher WA-1 pseudovirus neutralizing titers than the group immunized with S-2Pᵦ-TM (*p* = 0.0109). The neutralizing titers in the mouse sera following immunization with 10 μg of S-2P-NDVLP-1T (GMT ID_50_ value 321) were 1.7-fold higher than the sera following immunization with 10 μg of S-2P-TM (GMT ID_50_ value of 187), although not statistically significant. The pseudovirus neutralizing titers in the mouse sera following immunization with 50 μg of S-2P-NDVLP-1T (GMT ID_50_ value 619) were 1.9-fold higher than those of the 10 μg group, although not statistically significant. The pseudovirus neutralizing titers in the mouse sera following immunization with 50 μg of S-2P-TM were 1.2-fold higher than those of the 10 μg group, although not statistically significant (Figure 2D).

We next characterized Beta spike-specific IgG titers and Beta pseudovirus neutralizing titers. Consistent with the results obtained from analyses of WA-1 spike-specific antibody responses, after the second genetic delivery (week 6), Beta spike-specific IgG titers (endpoint GMT ranging from 4 × 10^4^ to 1 × 10^5^) increased by 8- to 20-fold compared with the IgG titers after the first genetic delivery at week 2, with endpoint GMT ranging from 2.5 × 10^3^ to 8.5 × 10^3^ (Figure 2E). Notably, Beta pseudovirus neutralizing titers observed in the mouse sera immunized with S-2Pᵦ-NDVLP-3T (GMT ID_50_ value 1775) were 4.5-fold higher than those immunized with S-2Pᵦ-TM (GMT ID_50_ value, 398) (*p* = 0.003) and 2.9-fold higher than those immunized with S-2Pᵦ-NDVLP-1T (GMT ID_50_ value, 603) (*p* = 0.022). Beta pseudovirus neutralizing titers in the mouse sera immunized with S-2Pᵦ-NDVLP-1T were 1.5-fold higher than those immunized with S-2Pᵦ-TM (Figure 2F), although not statistically significant. Similarly, Beta pseudovirus neutralizing titers in the mouse sera immunized with 50 μg of S-2P-NDVLP-1T (GMT ID_50_ value 583) were 2.6-fold higher than those immunized with 50 μg of S-2P-TM (GMT ID_50_ value 226), although not statistically significant, but significantly higher than those immunized with 10 μg of S-2P-NDVLP-1T (GMT ID_50_ value 102) by 5.7-fold (*p* = 0.0119). Beta pseudovirus neutralizing titers in the mouse sera immunized with 50 μg of S-2P-TM were 1.9-fold higher than the corresponding 10 μg group (GMT ID_50_ value 119), although not statistically significant (Figure 2F). Comparing neutralization of WA-1 versus Beta pseudovirus (Figure 2D,F), the S-2P-NDVLP-1T and S-2P_β_-NDVLP-1T 50-μg groups neutralized both WA-1 and Beta pseudoviruses equally well. However, the S-2P_β_-NDVLP-3T group achieved better neutralization of Beta than of WA-1, and the S-2P_β_-TM group showed greater neutralization of the Beta pseudovirus than did the S-2P-TM 50-μg group, although neutralization of the WA-1 pseudovirus was at similar levels in the corresponding groups.

### 3.3. Genetic Delivery of S-2P-NDVLP-1T Achieved the Highest Peak Neutralizing Titer, Whereas S-2Pᵦ-NDVLP-3T Produced a More Durable Neutralizing Response

**Figure 3:**
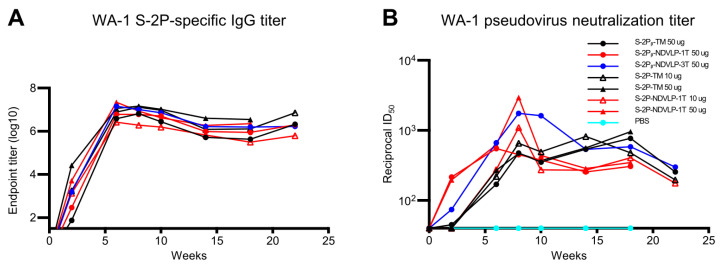
Genetic delivery extended the humoral immune responses in mice. (**A**) A general trend of antibody durability of WA-1 spike-specific IgG titers (area under curve). Sera samples were group-pooled. Genetic delivery of either of an eVLP platform or a protein subunit platform construct achieved a high IgG titer peak at week 6 and lasted to week 22. (**B**) A general trend of neutralizing antibody durability of WA-1 pseudovirus neutralizing titers. Genetic delivery achieved the highest neutralizing titer peak at week 8; moreover, the neutralizing titer peak in the S-2Pᵦ-NDVLP-3T-immunized group lasted from week 8 to 14. Genetic delivery of S-2Pᵦ-TM or S-2P-TM induced a moderate neutralizing titer peak, which appeared at week 8 and lasted to week 18 (See Figure S3 for neutralizing titers on individual animals from select groups).

Next, we evaluated whether the genetic delivery of eVLP could provide durable humoral immune responses in mice. The mouse sera were pooled by group at each bleeding time point (to reduce the overall samples for pseudovirus neutralizing testing) and assessed WA-1 SARS-CoV-2 spike-specific IgG titers and pseudovirus neutralizing titers over a course of 22 weeks. A general trend of antibody durability was observed as follows. The WA-1 spike-specific IgG titers for all immunized mouse groups peaked at week 6 and consistently extended to week 10, followed by a slight decline at week 14, then stayed at similar levels through week 22 (Figure 3A). The pseudovirus neutralizing titers for most immunized mouse groups peaked at week 8 and dropped at week 10 (Figure 3B).

The highest peak pseudovirus neutralizing titer was observed in the group-pooled sera immunized with 50 μg of S-2P-NDVLP-1T (ID_50_ value 2949) at week 8, which was 6.3-fold higher than the group-pooled sera immunized with 50 μg of S-2P-TM (ID_50_ value, 467). The second highest neutralizing titers were observed in the group-pooled sera immunized with 50 μg of S-2Pᵦ-NDVLP-3T (ID_50_ value 1750) at week 8, which was 3.7-fold higher than the group-pooled sera immunized with 50 μg of S-2Pᵦ-TM (ID_50_ value, 476). The pseudovirus neutralizing titer peak in the group-pooled mouse sera immunized with a low dose of S-2P-NDVLP-1T (ID_50_ value, 1096) at week 8 was 1.7-fold higher than the group-pooled mouse sera immunized with a low dose of S-2P-TM (ID_50_ value, 655). For the group-pooled mouse sera immunized with S-2Pᵦ-NDVLP-1T, the pseudovirus neutralizing titer (ID_50_ value, 547) peaked at week 6 and dropped at week 8. For three group-pooled mouse sera immunized with a high dose and a low dose of S-2P-TM, as well as with a high dose of S-2Pᵦ-TM, the pseudovirus neutralizing titers peaked at week 8 with similarly moderate levels, consistently extended through week 18, then dropped. The peaks of pseudovirus neutralizing titers induced with either S-2P-NDVLP-1T or S-2Pᵦ-NDVLP-1T rapidly dropped to moderate levels at week 10, followed by an extension to week 18 and a moderate drop at week 22. Notably, pseudovirus neutralizing titers peaked in group-pooled mouse sera immunized with S-2Pᵦ-NDVLP-3T, consistently extended from week 8 through week 10, then started two moderate drops at weeks 14 and 22, respectively. Overall, the eVLP constructs elicited higher neutralizing responses than the corresponding full-length spikes. The S-2Pᵦ-NDVLP-3T group also exhibited relatively strong and long-lasting neutralization responses.

To provide more definitive neutralization results, we measured neutralizing titers with individual serum samples collected at week(s) 0, 8, and 10 from four mouse groups immunized with 50 μg of S-2P-TM, S-2P-NDVLP-1T, S-2P_β_-NDVLP-3T, and control PBS. The results confirm the highest neutralizing titer at week 8 in the mouse group immunized with 50 μg of S-2P-NDVLP-1T (Figure S3B) and that the neutralizing titer peak in the mouse group immunized with 50 μg of S-2P_β_-NDVLP-3T can extend to week 10 (Figure S3C).

### 3.4. Genetic Delivery of S-2P-NDVLP-1T or S-2P-TM Induced Strong SARS-CoV-2 Spike-Specific T-Cell Responses

**Figure 4:**
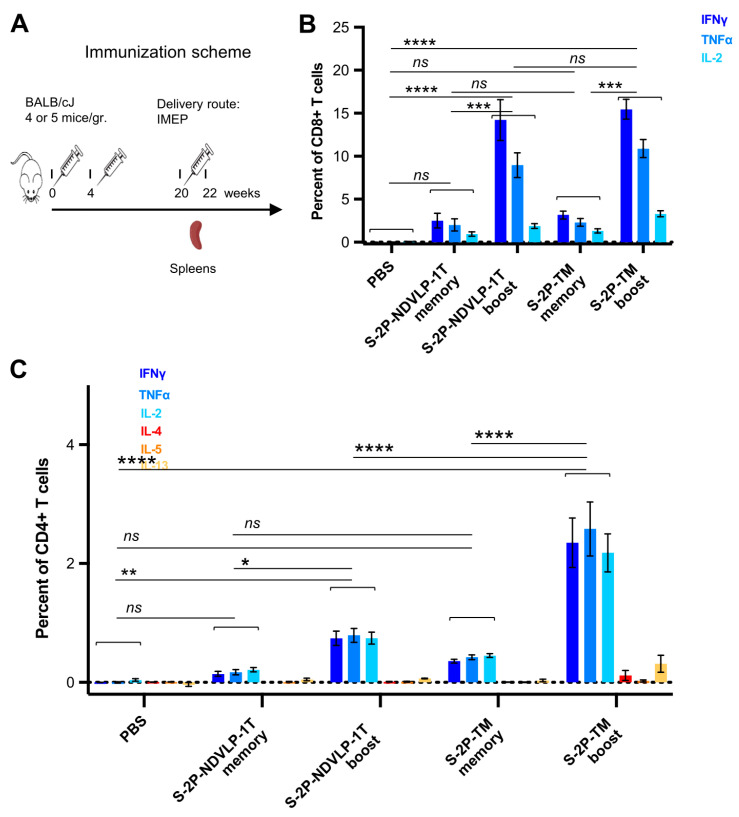
Genetic delivery generated strong anti-SARS-CoV-2 spike Th1-cellular immune responses. (**A**) Scheme of the genetic delivery regimen for splenic T-cell study. SARS-CoV-2 spike-specific CD4+ or CD8+ T cells in mouse spleens before and after the third genetic delivery were defined as memory or boost T cells. (**B**) Either S-2P-TM or S-2P-NDVLP-1T robustly activated a high percent of memory or boost spike-specific splenic CD8+ T cells expressing Th1 cytokines of IFNγ, TNFα, and IL-2. The percent of spike-specific splenic CD8+ T cells in boost groups was about three- to sevenfold higher than that in memory groups, and there was a statistical difference between the boost group and the memory group at *p* < 0.001. (**C**) Similarly, either S-2P-TM or S-2P-NDVLP-1T activated a high percent of memory or boost spike-specific splenic Th1 CD4+ T cells expressing Th1 cytokines of IFNγ, TNFα, and IL-2. The percent of spike-specific splenic Th1 CD4+ T cells activated by S-2P-TM in the boost group was about sixfold higher than that in the memory group at *p* < 0.0001 and also significantly higher than that in the 2P-NDVLP-1T group at *p* < 0.0001. The percent of spike-specific splenic Th1 CD4+ T cells activated by S-2P-NDVLP-1T in the boost group was about fourfold higher than that in the memory group at *p* < 0.05. Unbiased Th2 CD4+ T-cell response in (**C**), with no statistical difference among memory and boost groups expressing Th2 cytokines IL-4, IL-5, and IL-13. *p* values were designated as *: *p* < 0.05; **: *p* < 0.01; ***: *p* < 0.001; ****: *p* < 0.0001, and *ns*: not significant.

Next, T-cell responses and induction of systemic cytokines in mice were explored following the genetic delivery of a high dose (50 μg) of the DNA construct encoding S-2P-TM or S-2P-NDVLP-1T, with a control group of mice receiving PBS only. All groups of mice received three doses at week(s) 0, 4, and 20 (Figure 4A). We assessed SARS-CoV-2 spike-specific T cells from mouse spleens four months following the second genetic delivery (defined as memory T cells) or eleven days following the third genetic delivery (defined as boost T cells). Ficoll-isolated splenocytes were stimulated for 6 h with WA-1 SARS-CoV-2 spike peptides (S1 and S2 peptide pools), and Th1 cytokine production (IFNγ, TNFα, and IL-2) or Th2 cytokine production (IL-4, IL-5, and IL-13) was assessed as previously described [17]. Both CD4+ and CD8+ spike-specific T cells were detected four months after the second genetic delivery, and the third genetic delivery increased the frequency of CD4+ and CD8+ T cells expressing Th1 cytokines after peptide stimulation, indicating that genetic delivery stimulated both CD4+ and CD8+ T-cell responses to spike. While spike-specific CD8+ T cells were present in the spleen four months after the second genetic delivery, notably, the third genetic delivery boosted the frequency of responding CD8+ T cells by three- to sevenfold. The frequency of CD8+ T cells producing IFNγ in response to spike peptides reached about 10% of all splenic CD8+ T cells (Figure 4B). No significant difference existed in either memory or boosted CD8+ T-cell responses between S-2P-TM and S-2P-NDVLP-1T groups, but there was a significant difference between the boosted CD8+ T-cell group and the memory CD8+ T-cell group in the mice immunized with S-2P-TM or S-2P-NDVLP-1T (*p* < 0.001) (Figure 4B). Similarly, spike-specific splenic CD4+ T-cell responses were readily detected four months after the second genetic delivery, and the third genetic delivery increased the frequency of Th1-cytokine-producing CD4+ T cells in the spleens by four- to sixfold after peptide pool stimulation (Figure 4C). These CD4+ T-cell responses exhibited a Th1-dominant profile, and the percentage of Th1-cytokine-producing CD4+ T cells in either memory or boosted CD4+ T cells with the genetic delivery of S-2P-TM was twofold or threefold higher than that in association with the genetic delivery of S-2P-NDVLP-1T in parallel. Furthermore, there was a significant difference between the two boost groups at *p* < 0.0001 (Figure 4C), suggesting that genetic delivery of S-2P-TM activated more Th1-cytokine-producing CD4+ T cells than S-2P-NDVLP-1T. As expected, genetic delivery did not elicit Th2 cytokine-producing CD4+ T-cell responses, as indicated by an absence of increased Th2 cytokines, IL-4, IL-5, and IL-13 (Figure 4C). Overall, the results indicate a Th1-biased response in mice following genetic delivery of SARS-CoV-2 spike immunogens.

## 4. Discussion

The current study demonstrates that the profiles of humoral immune responses in animals achieved with the genetic delivery of a single-gene-transcript eVLP platform were comparable to those attained with the immunization of in vitro purified eVLPs in a previously reported study [6]. Comparison of genetic delivery of eVLP with the previously reported immunization of in vitro purified eVLP is summarized in Table 2. GMT comparison analysis shows the neutralizing titer (GMT of 4552) induced with the immunization of 50 μg of in vitro purified S-2P-NDVLP to be superior in terms of neutralization relative to the neutralizing titer (GMT of 2949) achieved with genetic delivery of the analogous S-2P-NDVLP-1T, although the ratio of in vitro purified S-2P-NDVLP-derived GMT (4552) to S-2P-derived GMT (1178) was comparable to the ratio of S-2P-NDVLP-1T-derived GMT (2949) to S-2P-TM-derived GMT (476). It is plausible that the GMT variability between these two eVLP delivery methods is due to differences in the amount of genetically delivered eVLPs and in vitro purified eVLPs. Analysis of in vitro eVLP expression revealed that the production of 50 μg of in vitro purified S-2P-NDVLP required approximately 200 μg of either S-2P-NDVLP-1T plasmid or S-2P-NDVLP-3T plasmid. Overall, after a single dose of immunization in mice, both eVLP delivery platforms elicited modest pseudovirus neutralizing titers, while their counterparts, protein subunits of S-2P-TM, S-2Pᵦ-TM, or S-2P, did not. After a second immunization in mice, both eVLP delivery platforms elicited higher pseudovirus neutralizing titers than their protein subunit counterparts. The genetic delivery of eVLPs resulted in a high peak of neutralizing titers but of shorter duration (Figure 3B). Moreover, genetic delivery of S-2Pᵦ-NDVLP-3T elicited a combination of stronger and longer-lasting pseudovirus neutralizing titers than S-2Pᵦ-TM or S-2Pᵦ-NDVLP-1T. The neutralizing titers induced by cell-membrane-anchored S-2P or S-2Pᵦ were modest and appeared later but lasted longer, as shown in Figure 3B. The observation that the Beta vaccine showed greater neutralization of the Beta pseudovirus than did the WT vaccine (50 μg; Figure 2) suggests a vaccine-to-viral strain correlation, as well as the antigenic specificity of these vaccines.

Genetic delivery bypasses the challenges of purification and instability of eVLP-based vaccines in vitro [3,4,10,18,29], enabling the use of eVLP-based vaccines for the vaccination of large populations, although the results obtained in mice need to be confirmed in non-human primates (and humans) [30]. However, it remains to be determined whether the eVLP-based vaccines described here can offer sterilizing immunity against SARS-CoV-2 infection.

In addition, the genetic delivery of a DNA construct of S-2P-TM or S-2P-NDVLP-1T generated high spike-specific T-cell responses. Recent studies have shown that SARS-CoV-2-specific T-cell responses are essential for viral clearance, provide robust memory, mediate recognition of viral variants, and may effectively prevent initial viral infection in concert with antibody responses [31]. In the current study, the frequency of spike-specific T cells after DNA immunization was higher than that previously reported in mice immunized with Moderna mRNA-1273 [17,32]. This finding is consistent with the clinical observation that robust T-cell responses were induced after dual mRNA vaccination [33,34]. This observation may provide additional insight into the role of T-cell responses after genetic delivery in protection against SARS-CoV-2 infection.

**Figure 5:**
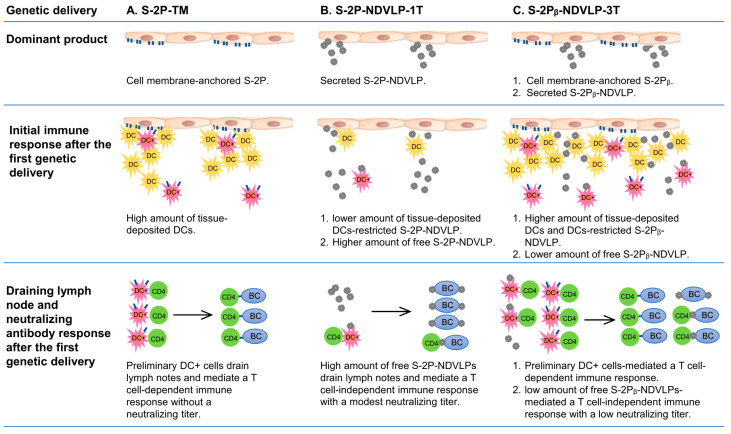
Illustration of mechanistic modes and special features of each vaccine platform in inducing neutralizing immunity after the first genetic delivery. DC, dendritic cells; DC+, spike-specific dendritic cells; BC, B cells.

The current study also intended to examine the special features inducing neutralizing immunity through various vaccine platforms, specifically the humoral immune responses achieved by the genetic delivery of a single-gene-transcript eVLP platform of S-2P-NDVLP-1T/S-2Pᵦ-NDVLP-1T, of a multiple-gene-transcript eVLP platform of S-2Pᵦ-NDVLP-3T, and of a protein subunit platform of S-2P-TM or S-2Pᵦ-TM in mice. Based on the presented results and prior knowledge [4,35,36,37,38,39,40], we discussed the putative mechanistic models and special features of each vaccine platform in inducing neutralizing immunity after the first genetic delivery (Figure 5).

Cell-membrane-anchored S-2P or S-2Pᵦ functions as an immobile particle antigen, while S-2P-NDVLP or S-2Pᵦ-NDVLP functions as a mobile particle antigen. The genetic delivery of S-2P-TM or S-2Pᵦ-TM was unable to elicit a pseudovirus neutralizing titer after the first genetic delivery at week 2 (Figure 2C) but able to induce higher levels of spike-specific CD4+ T cells in lymphoid tissues, such as in the spleen (Figure 4C). Thus, the levels of spike-specific CD4+ T cells reflect the levels of spike-specific DCs (DC+), and membrane-anchored S-2P or S-2Pᵦ on the muscle cell surfaces localizes a considerable amount of DCs in muscle tissue, where tissue-localized DCs interact with membrane-anchored S-2P or S-2Pᵦ and are activated as spike-specific DCs (DC+). The spike-specific DCs migrate into lymphoid tissues and activate CD4+ T cells as spike-specific CD4+ T cells, which then trigger CD4+ T-cell-dependent humoral immune response (Figure 5A). The mechanism resulting in spike-specific DCs/CD4+ T-cell response being unable to elicit an observed neutralizing titer after the first genetic delivery remains to be determined. In contrast, eVLPs, as mobile particles, directly migrated into lymph nodes, activated B cells, and led to modest pseudovirus neutralizing titers after the first genetic delivery at week 2, suggesting a T-cell-independent humoral immune response (Figure 5B). The genetic delivery of S-2Pᵦ-NDVLP-3T dominantly produced both mobile particles of S-2Pᵦ-NDVLP and immobile particles of cell-membrane-anchored S-2Pᵦ in muscle tissue. The observation of relatively low pseudovirus neutralizing titers after the first genetic delivery of S-2Pᵦ-NDVLP-3T at week 2 (Figure 2C) inspired us to speculate that cell-membrane-anchored S-2Pᵦ results in a higher amount of tissue-localized DCs, which restrict further S-2Pᵦ-NDVLPs in the muscle tissue. In turn, a lower amount of free S-2Pᵦ-NDVLPs migrates into lymph nodes, resulting in a low pseudovirus neutralizing titer (Figure 5C). It is also possible that more potent neutralizing titers were achieved due to the spike S-2P antigen being expressed in a “natural viral membrane” fully conformational state (like “authentic” form) on the eVLPs, while the membrane-anchored S-2P may not have been in a fully native form on the host cell surfaces. All these possibilities remain to be determined in further studies.

The second genetic delivery of S-2Pᵦ-NDVLP-3T robustly boosted both cell-membrane-anchored, S-2Pᵦ-mediated, and S-2Pᵦ-NDVLP-mediated humoral immune responses, which correlate with strong and long-lasting pseudovirus neutralization titers (Figure 2F and Figure 3B). This observation may imply that combined immunization with two different platform vaccines can synergically achieve the best humoral immune responses. In the current study, S-2Pᵦ-NDVLP-3T produced two platform vaccines in vivo—cell-membrane-anchored S-2Pᵦ and S-2Pᵦ-NDVLP—which may be functionally similar to combined immunization with S-2Pᵦ-TM and S-2Pᵦ-NDVLP-1T. Such observations have also been reported with heterologous vaccination with both ChAdOx1 and mRNA1273, which achieved expanded and enhanced humoral immune responses [41]. The results achieved in the current study not only help us to better understand the genetic delivery of different vaccine platforms, resulting in different immunogenicity outcomes, but also provide a practical strategy for eVLP-based vaccine development that should be simple, rapid, scalable, and efficient for the vaccination of large populations.

## 5. Conclusions

Our study demonstrates that the neutralization results achieved with the genetic delivery of a single-gene-transcript eVLP platform in mice are highly comparable to those previously reported with respect to immunization with in vitro purified eVLPs [6]. Furthermore, the results highlight that the genetic delivery of a multiple-gene-transcript eVLP platform vaccine can elicit strong and long-lasting anti-SARS-CoV-2 neutralization, that DNA immunization can induce notably high T-cell responses, and that different vaccine platforms may have different mechanisms for inducing neutralizing antibodies.

## 6. Patents

US Patent Application 63/140,250 filed 21 January 2021, entitled “Newcastle disease virus-like particle displaying prefusion-stabilized SARS-CoV-2 spike and its use”.

## Figures and Tables

**Table 1:** DNA constructs used in animal immunization.

DNA Immunogen	Description
S-2Pᵦ-TM	Protein subunit vaccine, producing cell membrane-anchored prefusion-stabilized SARS-CoV-2 spike (Beta variant)
S-2Pᵦ-NDVLP-1T	Single-gene-transcript vaccine, producing unique S-2Pᵦ-NDVLP (Beta variant)
S-2Pᵦ-NDVLP-3T (S-2Pᵦ-NDV-Ftm, NDV-M, and NDV-NP)	Multiple-gene-transcript vaccine, with cogenetic delivery of NDV-M, NDV-NP, and S-2Pᵦ-NDV-Ftm (Beta variant), producing both cell-membrane-anchored S-2Pᵦ and S-2Pᵦ-NDVLP; used in comparison with S-2Pᵦ-NDVLP-1T
S-2P-TM	Protein subunit vaccine, producing cell-membrane-anchored prefusion-stabilized SARS-CoV-2 spike (WA-1 variant), with an amino acid sequence identical to that of the Moderna or Pfizer mRNA vaccine; used as a control for S-2Pᵦ-TM
S-2P-NDVLP-1T	Single-gene-transcript vaccine, producing unique S-2P-NDVLP (WA-1 variant); used as a control for S-2Pᵦ-NDVLP-1T

**Table 2:** Comparison of the genetic delivery of eVLP with published immunization of in vitro purified eVLP [6].

Delivery Platform	In Vitro Purified Immunogen ^(a)^		Genetic Delivery of DNA Construct			
Immunogen platform	Protein subunit	eVLP	Protein subunit	eVLP	Protein subunit	eVLP
Immunogen	S-2P	S-2P-NDVLP	S-2P-TM	S-2P-NDVLP-1T	S-2Pᵦ-TM	S-2Pᵦ-NDVLP-1T
Dosage	2 µg ^(b)^	50 µg ^(b)^	50 µg	50 µg	50 µg	50 µg
Single dose. S-2P-specific IgG titer (log_10_)	4.0987 (week 2)	4.1853 (week 2)	3.1117 (week 2)	3.1517 (week 2)	4.0820 (week 2)	3.8374 (week 2)
Two doses. S-2P-specific IgG titer (log_10_)	5.8343 (week 5)	4.9193 (week 5)	5.0316 (week 6)	4.7230 (week 6)	4.5993 (week 6)	4.6303 (week 6)
Single dose. Pseudovirus neutralizing titer (ID_50_, GMT)	No titer (threshold) (week 2)	172 (week 2)	No titer (threshold) (week 2)	196 (week 2)	No titer (threshold) (week 2)	215 (week 2)
Two doses. Pseudovirus neutralizing titer (ID_50_, GMT)	1178 (week 5)	4552 (week 5)	476 (week 8)	2949 (week 8)	170 (week 6)	547 (week 6)

^(a)^. Data were adopted from a prior publication [6]. ^(b)^. According to antigenic quantitation with ELISA in a previously reported study [6], 2 µg of S-2P soluble protein is roughly equal to 50 µg of S-2P-NDVLP or more.

## Data Availability

The resources and materials used in the manuscript are available from the corresponding author upon request. Any such requests should be directed to and will be fulfilled by Peter D. Kwong (pdkwong@nih.gov).

## Supplementary Materials

The following supporting information can be downloaded at: https://www.mdpi.com/article/10.3390/vaccines11091438/s1, Figure S1. Illustration of single-gene-transcript technology that enables in vivo production of unique eVLPs in the same cell versus multiple-gene-transcript technology that produces both eVLPs and membrane-anchored spikes among different cells in vivo. Figure S2. In vitro expression characterization of genetic DNA constructs. Figure S3. Individual neutralizing titers within selected groups.
