# Supplementary material for: Enhancing Anti-SARS-CoV-2 Neutralizing Immunity by Genetic Delivery of Enveloped Virus-like Particles Displaying SARS-CoV-2 Spikes
Source: Vaccines (Basel). 2023 Aug 31;11(9):1438. doi: 10.3390/vaccines11091438 (PMC10537688; doi:10.3390/vaccines11091438)
Supplement: Supplementary file 1 [file vaccines-11-01438-s001.zip › vaccines-2529503-supplementary.pdf]

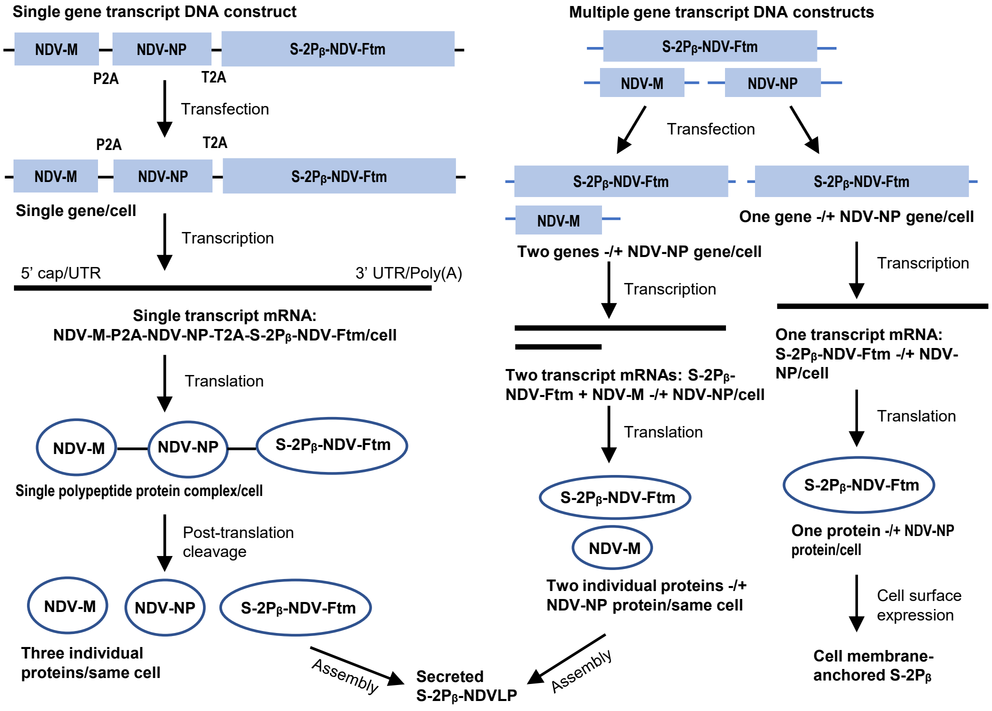

Figure S1. Illustration of single gene transcript technology that enables *in vivo* production of unique eVLPs in the same cell versus multiple gene transcript that produce both eVLPs and membrane-anchored spikes among different cells *in vivo*.

Figure S2

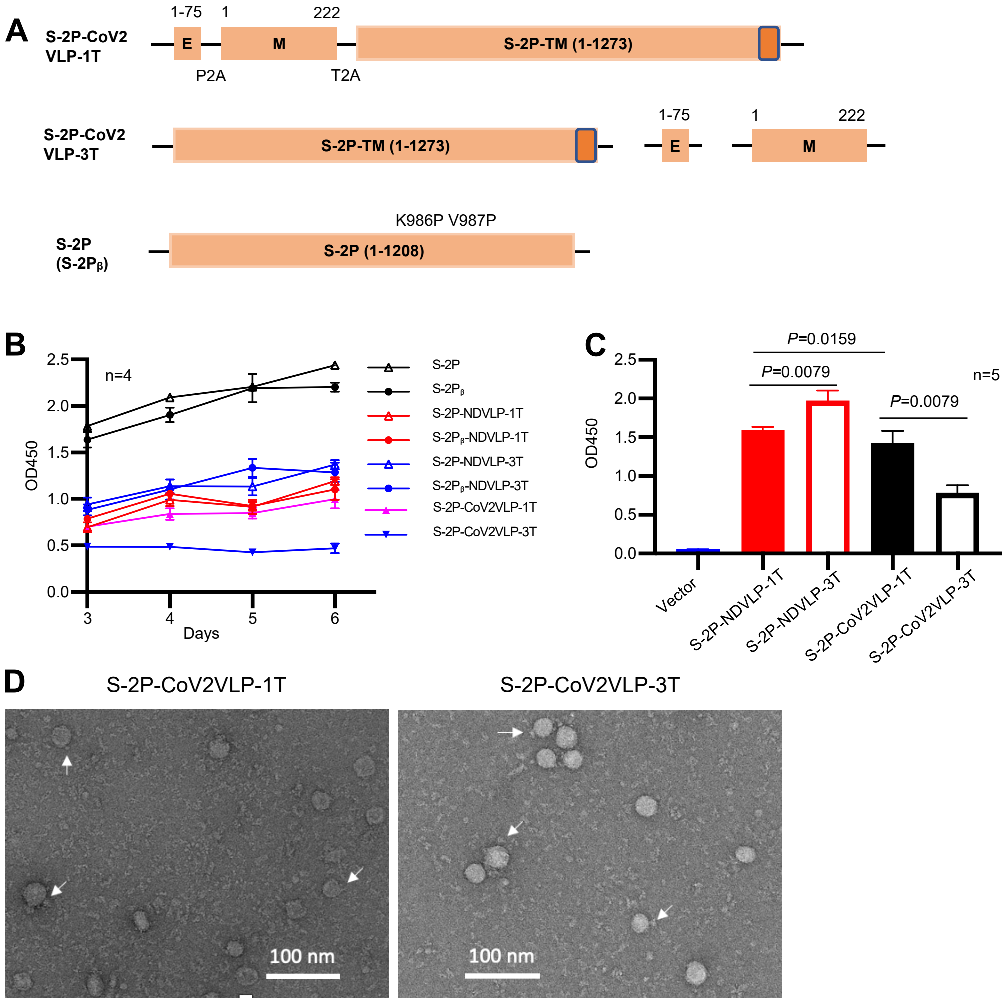

Figure S2. *In vitro* expression characterization of genetic DNA constructs. (A) Schematic generation of genetic DNA constructs encoding SARS-CoV-2 virus-like particles displaying prefusion-stabilized WA-1 spikes (S-2P-CoV2VLPs). Top linear diagram represents a single gene transcript DNA construct (S-2P-CoV2VLP-1T), three WA-1 SARS-CoV-2 genes encoding envelope protein (E), matrix protein (M) and a full length of prefusion-stabilized spike (S-2P-TM) linked by two self-cleaving peptide sequences P2A and T2A, respectively. Middle linear diagrams represent multiple gene transcript DNA constructs (S-2P-CoV2VLP-3T) encoding E, M and S-2P-TM, respectively. Bottom linear diagram represents two DNA constructs encoding soluble S-2P or S-2P$_\beta$, prefusion-stabilized spike ectodomain. (B) Expression characterization of various eVLP genetic DNA constructs. eVLP yield (OD$_{450}$) vs. harvest time (days) measured by eVLP ELISA with an antibody S309. (C) Various eVLP genetic DNA constructs were further characterized by eVLP-binding ELISA with an antibody S309. Two-tailed Mann-Whitney *t* test was used to determine the significant difference between the groups. (D) Structural and morphological characterization of S-2P-CoV2VLPs by negative staining transmission electron microscopy, showing well-defined S-2P-CoV2VLP (20-40 nm) with scattered spikes (~10 nm) on the surfaces of eVLPs.

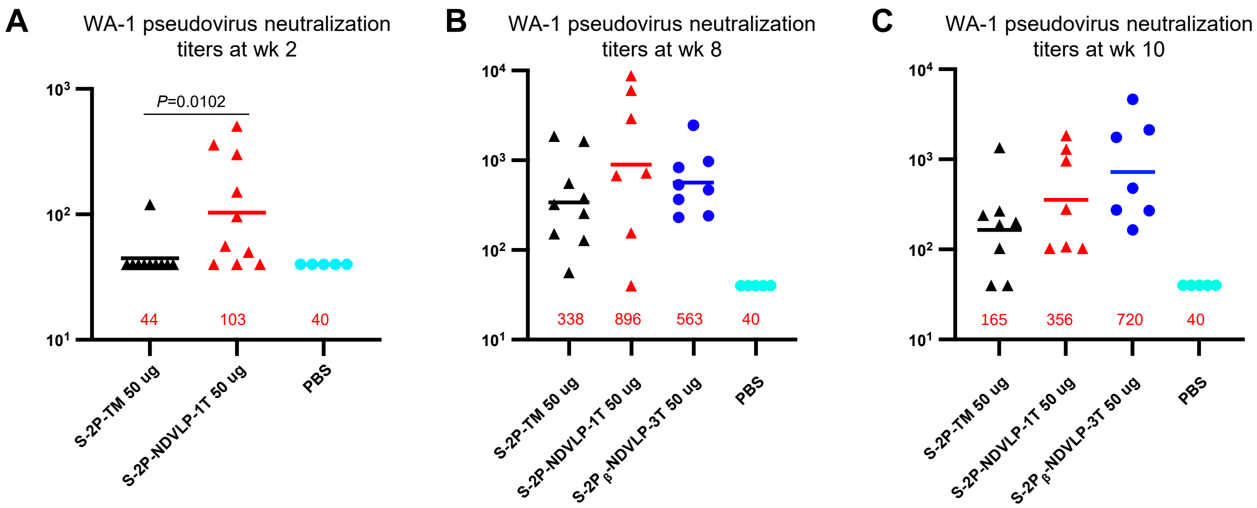

Figure S3. Individual neutralizing titers within selected groups. (A) The neutralizing titers after the first genetic delivery at week 2 were assessed with individual serum samples from three mouse groups immunized with 50 μg of S-2P-TM, S-2P-NDVLP-1T and control PBS, respectively. S-2P-NDVLP-1T elicited a modest pseudovirus neutralizing titer. (B) The week-8 neutralizing titers after the second genetic delivery, assessed with individual serum samples from four groups immunized with 50 μg of S-2P-TM, S-2P-NDVLP-1T, S-2P$_{\beta}$-NDVLP-3T and control PBS, respectively, showed the highest neutralizing titer peak achieved in the group of S-2P-NDVLP-1T, although no statistically significant difference among the S-2P-TM, S-2P-NDVLP-1T, and S-2P$_{\beta}$-NDVLP-3T groups. (C) The neutralizing titer results at week 10, assessed with individual serum samples from four groups immunized with 50 μg of S-2P-TM, S-2P-NDVLP-1T, S-2P$_{\beta}$-NDVLP-3T and control PBS, respectively, showed higher neutralizing titers achieved in the S-2P$_{\beta}$-NDVLP-3T group, although no statistically significant difference among the S-2P-TM, S-2P-NDVLP-1T, and S-2P$_{\beta}$-NDVLP-3T groups was observed. Two-tailed Mann-Whitney test was used to calculate statistical differences. Some individual serum samples did not survive storage and were excluded from the assays performed in (B) and (C).
